# Pupils’ Use of Social Media and Its Relation to Mental Health from a School Personnel Perspective: A Preliminary Qualitative Study

**DOI:** 10.3390/ijerph18179163

**Published:** 2021-08-31

**Authors:** Gunnhild Johnsen Hjetland, Viktor Schønning, Bodil Elisabeth Valstad Aasan, Randi Træland Hella, Jens Christoffer Skogen

**Affiliations:** 1 Department of Health Promotion, Norwegian Institute of Public Health, 5015 Bergen, Norway; Viktor.Schonning@fhi.no (V.S.); Jens.Christoffer.Skogen@fhi.no (J.C.S.); 2 HUNT Research Centre, Department of Public Health and Nursing, Faculty of Medicine and Health Sciences, Norwegian University of Science and Technology, 7600 Levanger, Norway; Bodil.E.V.Aasan@ntnu.no; 3 Department of Work, Social Services and Housing, Section for Children, Families and Disabled, 5020 Bergen, Norway; Randi.Hella@bergen.kommune.no; 4 Alcohol and Drug Research Western Norway, Stavanger University Hospital, 4068 Stavanger, Norway; 5 Department of Public Health, University of Stavanger, 4036 Stavanger, Norway

**Keywords:** social media, mental health, adolescents, focus group study

## Abstract

The extent of mental health problems among adolescents seems to be on the rise, and this observed trend has often been linked to a coinciding increase in social media use. The goal of the current preliminary study was to investigate how senior high school personnel experience the role of social media in relation to the mental health of their pupils. Two focus group interviews (total n = 11) were completed and analyzed using reflexive thematic analysis, resulting in 4 themes and 11 subthemes. The results illustrate that school personnel experience social media as a tool for communication, but also as a potential cause of mental health issues and reduced academic performance among pupils. The participants called for schools to become better equipped to meet the opportunities and challenges of social media.

## 1. Introduction

Over the course of a few decades, social media has come to play an important role in most people’s daily lives. Using the definition by Kietzmann et al. ([1], p. 1), social media “employ mobile and web-based technologies to create highly interactive platforms via which individuals and communities share, co-create, discuss, and modify user-generated content”. Thus, social media entail a wide range of activities and purposes, from social networking to sharing creative content and online gaming, using a multitude of different platforms. Adolescents are particularly active users, with almost half saying they are “almost constantly” online [2,3]. Among Norwegian pupils in senior high school, 47 % report spending more than two hours on social media every day [4].

The increased use of social media among adolescents over the last decades has been suggested as a potential cause of the increase in reported mental health problems among adolescents, as the phenomena coincide [5,6,7,8]. In the public discourse, social media is largely portrayed as having a negative influence on young people’s mental health [9], with headlines such as “Social media damages teenagers’ mental health, report says” [10] and “Anxiety on the rise among the young in social media age” [11]. However, within the research field, there is a discussion regarding the impact and relevance of social media in relation to adolescents’ mental health and well-being. While some studies have identified a statistically significant association between increased social media use and mental health problems (e.g., [7,12,13]), the associations are small and their practical consequences have been questioned [14,15]. To illustrate this point, Orben and Przybylski [15] demonstrated that the negative effect of social media use on subjective well-being was similar to the effect of eating potatoes, and also that wearing glasses had a 1.5 times larger effect than social media use. Importantly, the quantitative research available has some key weaknesses, where many of the studies are based on relatively simple measures of time spent on social media or the frequency of use [16,17]. The relationship between social media and mental health is seemingly very complex, and the impact of social media use likely depends on the specific activities people engage in on social media, who they interact with, how invested they are, their underlying motives, and their personal characteristics [18,19]. Furthermore, most studies are cross-sectional, investigating the association between social media use and mental health at only one point in time and where no causation can be established [16].

Qualitative studies enable an in-depth exploration of individuals’ experiences and can nuance and give meaning to divergent quantitative findings [20,21]. Qualitative studies involving adolescents have shown that they experience both positive and negative effects of social media, and even the same activities on social media can give rise to both positive and negative emotions [22,23,24]. For example, Weinstein [24] showed that communication through social media could lead both to a feeling of closeness and to a feeling of isolation, and that other people’s posts (photos, videos, etc.) were entertaining and led to admiration, but also to envy and self-awareness about their own appearance. Furthermore, several studies have shown that keeping in touch with friends and participating in the digital social arena is highly valued by users and constitutes an important motivation for using social media [23,24].

To date, both quantitative and qualitative enquiries into the relationship between social media and mental health rely on self-report. To our knowledge, no previous studies have examined the connection between social media and mental health from the perspective of school personnel (e.g., teachers and other tuition personnel interacting daily with pupils). Adolescents spend a large part of their day-to-day life at school, and although adolescents may not discuss their social media use with their teachers, teachers and other school personnel are in a unique position to provide insight into how young people are affected by social media. They observe the pupils regularly over time and many have experience with several school cohorts who have used social media to varying degrees. In addition, teachers and other school personnel are important role models for pupils, and their attitudes and thoughts about social media may affect adolescents in important ways. Furthermore, the school context is central to preventive and health-promoting work, and school staff constitute an important resource in this respect [25,26]. In line with this, it is important to know how school staff regard the role of social media in the lives of adolescents. In this preliminary study, we aimed to explore school staff’s experiences and understandings of the relationship between social media use and adolescents’ mental health and well-being.

## 2. Materials and Methods

The study was a preliminary exploratory qualitative study using a focus group methodology [27,28,29]. The study is part of a larger project that aims to better understand the relationship between different aspects of social media use and mental health and well-being among adolescents using both qualitative and quantitative approaches. Further information about the project is available from previous publications [30,31].

### 2.1. Study Participants and Setting

Two groups of participants were recruited from two separate high schools in Bergen, Norway. The number of focus groups was set a priori and reaching saturation was deemed non-relevant due to the highly exploratory and preliminary nature of the study [32]. A contact person at each school recruited participants to the focus groups. Information about the study was given to all employees, and those interested approached the contact person. There was no previous relationship between the research personnel and the participants.

### 2.2. Data Collection

The focus groups met once for about 90 min with a ten-minute break halfway through the interview. The interviews were conducted at the schools during the autumn of 2019. The interview guide (see Supplementary Material S1) was adapted from a piloted interview guide for adolescents [30] and had two main questions:

1. How do you feel that social media can be a positive factor in the lives of adolescents?
2. How do you feel that social media can be a negative factor in the lives of adolescents?

Each of the main questions had several predefined follow-up questions that could be used by the moderator if necessary, and the moderator could also ask other ad-hoc questions deemed relevant during the interviews. In addition, the guide had one opening question and two closing questions. A clinical psychologist (GJH) and a Bergen municipality advisor with a master’s degree in health and welfare management (RTH) conducted the interviews. They took turns leading the interviews (moderator) and documenting the progress of the interview and non-verbal events (secretary). The participants explicitly agreed to the audio recording of the interviews. Following the interviews, the audio recordings were transcribed and de-identified into written form for analysis. Audio recordings were deleted after complete transcription.

### 2.3. Data Analysis

The interviews were analyzed using reflexive thematic analysis [33,34], which is a flexible analysis method for identifying, analyzing, and reporting patterns or themes in qualitative data. The analysis followed the six phases as proposed by Braun and Clarke [34]:

- Phase 1: Three of the authors (GJH, RTH, and BEVA) read and re-read the material and noted potential themes.
- Phase 2: The same three authors identified relevant text segments and created codes individually. Subsequently, the suggested codes were discussed in plenary and a final list of codes was made. BEVA used the agreed-upon codes to code the transcripts. The coding was performed in Nvivo, version 12 (QSR International, Melbourne, Australia).
- Phase 3: GJH, RTH, BEVA, and JCS independently sorted the codes into potential topics. VS provided an external perspective on the themes, following which we agreed on a set of topics.
- Phase 4: BEVA took the main responsibility for reviewing the topics in relation to the coded text segments and the entire data set (the transcribed interviews). The themes were discussed in face-to-face meetings with GJH, RTH, VS, and JCS.
- Phase 5: When a consensus about the themes was reached, the themes were collaboratively named and defined by all the authors.
- Phase 6: BEVA prepared the first draft, which was further processed and completed under the leadership of GJH and in collaboration with all the authors.

As in previous publications from the project, the author group endeavored to adhere to the approach put forward by Braun and Clarke [30], and no deviation from the above description was observed during data analysis.

### 2.4. Ethical Considerations

It was voluntary to participate in the focus group interviews. Informed consent was obtained from all subjects involved in the study at the time of the interview, after being informed about the purpose and procedures of the study, as well as how data would be handled in a secure way. Information was given orally and in writing. Participants were informed that the interviews would be audio recorded during recruitment, orally at the time of the interview, and in writing in the informed consent form. In addition, all participants explicitly agreed that the interview would be recorded prior to starting the recorder in each focus group. When the interviews were transcribed, all participants’ names were replaced with fictitious names, and any identifiable information was omitted or replaced.

## 3. Results

A total of seven men and four women between the ages of 24 and 62 (mean age 42 years) participated, all of whom were employed as teachers or welfare or pupil support staff with direct pupil contact. The participants had worked in senior high school for a mean of 10.2 years, with a median of 7 years and a range of 5 to 29 years. Table 1 provides an overview of the participants.

### Findings

**Figure 1:**
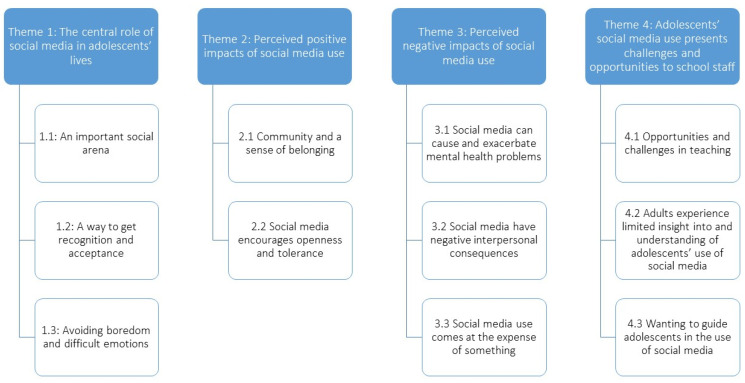
Overview of themes and sub-themes.

Based on the interviews, we created four main themes: (1) The central role of social media in adolescents’ lives, (2) Perceived positive impacts of social media use, (3) Perceived negative impacts of social media use, (4) Adolescents’ social media use presents challenges and opportunities to school staff (see Figure 1).

*Theme 1: The central role of social media in adolescents’ lives*

In both focus groups, the participants discussed how important social media seems to be for adolescents and pointed to several potential reasons for this.

Subtheme 1.1: An important social arena

Communication with peers was considered a key motivation as to why adolescents use social media. According to the participants, social media is a meeting place where adolescents can be part of a larger community and feel part of their peer group. But this also means that they have to be on social media in order to keep up and be where it happens.

*I think the word there is community. They feel that, that’s where they communicate, that’s where they are. And if they are not there, then they may be the only one in their class who is not there. And that isolates them very much. (FG2F1)*

Furthermore, one participant pointed out that social media seem to create a mutual expectation of being available at all times.

Subtheme 1.2: A way to get recognition and acceptance

In both focus groups, the participants highlighted adolescents’ need for recognition and acceptance, and that self-presentation through social media can cover this need in the form of “likes” and comments.

*I think that there is a basic psychological need that can be fulfilled by social media, for example when you talk about likes, then, that is about acceptance and recognition. (FG1M1)*

Subtheme 1.3: Avoiding boredom and difficult emotions

In one of the interviews, the participants argued that adolescents use their smartphones to escape loneliness and boredom, and also to avoid people’s gaze or avoid looking lonely in public.

*It is the need for safety. The phone is a safe space for them, because then they don’t have to meet others’ gaze when they are on the bus, for example. Then they can look at their phone instead. (FG1F2)*

Similarly, the participants believed that the phone is used as an escape route and a diversion from negative emotions. This strategy could then backfire when the adolescents put down their phone before going to bed.

*Theme 2: Perceived positive impacts of social media use*

The participants in both focus groups addressed perceived positive aspects of social media use.

Subtheme 2.1: Community and a sense of belonging

According to the participants, social media is an important social arena where young people can get a sense of belonging. They placed special emphasis on how pupils with poor social skills can find their social networks through online gaming or other social media.

*For those who have previously lived a very isolated and secluded life, mostly alone in their room, then social media may be sort of a crack in the wall where a little light comes in. (FG1M1)*

Subtheme 2.2: Social media encourages openness and tolerance

Furthermore, the participants talked about how social media can open for greater diversity and tolerance. They believed that subcultures become more visible and normalized through social media and that this enables people to be different. On social media, everyone can participate, regardless of who they are or what limitations they have:

*It is an arena where, or… where they meet each other. And in there, they’re all the same, in a way. They don’t see each other, it’s just “that person there”, in a way. If you understand. You kind of don’t see that “he’s in a wheelchair” or “she looks like that”. It’s just “what are your thoughts?” instead of. (FG2M1)*

*Theme 3: Perceived negative impacts of social media use*

Although the participants were asked about both negative and positive aspects of social media, negative aspects dominated the discussions, and the participants in both focus groups discussed how social media had a negative impact on adolescents.

Subtheme 3.1: Social media can cause and exacerbate mental health problems

According to participants, social media can lead to and exacerbate mental health problems. The participants highlighted upward social comparison as an important mechanism through which social media can have a negative impact on adolescents’ self-image. They believed social media provide adolescents with unrealistic role models and expectations that lead to body pressure and a desire to be “successful”:

*I think that living up to the image they really want of themselves, it does something with their mental state, because there are lots of influencers who tell you how to eat, what to eat, how to exercise, what to… and it becomes difficult to live up to it. It can affect their self-image, I think. (FG1F2)*

According to the participants, adolescents create a facade on social media and try to be something more than they are. The participants highlighted that it is completely natural to want to present oneself in the best possible way, but that it is unhealthy when the facade does not match with reality. One participant pointed out how vulnerable adolescents are, and that a lack of feedback or negative feedback on social media can affect how they feel. An example was how many “likes” they get (or do not get). The participants also discussed how social media use can cause stress. For example, one participant talked about how social media exacerbates the expectations adolescents have to themselves and others: That they should perform well both at school and in various leisure-time activities, while also having an exciting and fulfilling social life. In addition, the participants highlighted how social media creates an expectation to be constantly available, which interferes with homework or concentrating at school, leading to a persistent stress load.

Sub-theme 3.2: Social media have negative interpersonal consequences

In both focus groups, it was pointed out that social media have negative interpersonal consequences. Communication through social media was described as inferior to face-to-face communication. Some of the participants thought that the language on social media seemed colder and less empathetic, and that this was a consequence of not seeing the recipient and their emotional reactions. In particular, the participants felt that anonymity on social media makes it easier to be rude to others:

*And I feel that, it seems as if more and more people have less empathy. People no longer care about what those at the other end receive or how they express themselves. And the things they say online that they would never say face-to-face. (FG2M3)*

One participant described how this online language becomes part of everyday speech.

Furthermore, the participants described how social media potentially reinforce negative emotions when adolescents seek out groups of like-minded people online and encourage each other’s negative emotions and action patterns.

They also considered it easier to exclude others on social media because adults do not have access and you are not held responsible for what you do to the same extent as in face-to-face interactions. Furthermore, it was pointed out that exclusion becomes very visible on social media, and bullying or conflicts can be exacerbated because it is public, and others may join in. Not being included in closed groups was also mentioned as a negative aspect of social media.

*I think that what you are describing is not something new; it is not something that has occurred due to social media. But it’s just that now it is more visible. For us too, there was exclusion, there were people who were excluded and people who were included. But now it is manifested through social media. (FG1M1)*

Sub-theme 3.3: Social media use comes at the expense of something

Participants in both groups talked about how addictive social media are, and that young people spend too much time on social media. According to the participants, the extensive use of social media comes at the expense of creativity, the ability to concentrate, and social skills. For example, participants mentioned that adolescents try to do homework and use social media at the same time. One participant was concerned that social media weakens young people’s ability to delay gratification and their ability to concentrate, and that this could have major consequences for schooling and education. The same participant feared that social media could reinforce individual differences in the ability to concentrate, in that those who manage to put away their phones get to train their “cognitive stamina” to a greater extent than those who do not. Furthermore, some of the participants expressed concerns that extensive use of social media could lead to poorer oral language skills and poorer social skills due to less frequent face-to-face interaction:

*But I worry about what they miss when they are reading body language, for example. When they don’t, uh, talk to each other anymore, everything goes through social media. (FG1F2)*

The participants also discussed how adolescents use social media in the evening and get insufficient sleep, further contributing negatively to potential mental health issues.

*Theme 4: Adolescents’ social media use presents challenges and opportunitites to school staff*

In both groups, the participants discussed how they themselves are affected by adolescents’ use of social media, both in their own professional role, and some also as parents.

Subtheme 4.1: Opportunities and challenges in teaching

In both of the focus groups, the participants said that they have used social media in their teaching. One participant explained that it is easier to get feedback from pupils on Snapchat than on the school’s digital learning platform. Another participant found that the pupils were more personal on social media, thus providing insight into how they were doing.

Furthermore, several of the participants had noticed that the pupils used social media as a source of information. They worried that adolescents are too uncritical of information from the internet and social media, and argued that adolescents are particularly vulnerable to fake news and social media ‘echo chambers’. One of the participants suggested that the school should become better equipped for a world where all (mis)information is available, and rather teach pupils to be creative and source-critical:

*But I think that perhaps the solution would be to have, in a way, tasks that take into account that the information is there and is easily accessible, but that they have to use creativity and use their own experiences and reasoning to be able to answer the assignments. (FG6M3)*

Participants also discussed how social media disrupt teaching. One described that the pupils have their phone in their hand all the time, and that they struggle to pay attention to what is happening in class. Another participant found it difficult to achieve peace and quiet in the classroom:

*Because some students focus too much on it. If it’s not gaming, it’s the phone. And it is very challenging for us to create a calm and peaceful atmosphere in the classroom. (FG1F2)*

Furthermore, the participants found it challenging to relate to the fact that the pupils have access to their cellphone in the classroom and they had different strategies for solving this:

*FG1M1: But it is also not allowed to use the phone during class*

*FG1M3: No, that’s true. But what do we do then?*

*FG1M1: Yes, no, that…*

*FG1F2: Many of them are used to these “phone hotels” from junior high school, and then they come here and have access to it all the time, right.*

*FG1M1: Yes, yes.*

*FG1M2: When we made the class rules, we agreed that they should use flight mode during class.*

*FG1M3: Yes… but do they do it?*

*FG1M2: Yes. Always.*

Subtheme 4.2: Adults experience limited insight into and understanding of adolescents’ use of social media

Several of the participants said that they find it difficult to understand the adolescents’ use of social media and how much value they seem to attribute to social media:

*Most of them would probably trade a ‘like’ for a hug if they could. And that is a bit, like… Yes, I find it strange that they prioritize spending their time striving to be perfect on a screen rather than caring about how they are really doing in their lives. I think that’s a bit scary. (FG2M2)*

Furthermore, the participants described social media as a sanctuary where young people can be by themselves without adults. The downside of this was that adults have little access to what the adolescents are doing, and the participants worried about negative experiences and increased access to drugs and people with ill intentions. One of the participants called for parents to be more involved in what their children do on social media:

*Because they don’t have a clue themselves. Nor have they taken the time to get acquainted with it [social media]. And then they are not able to assess the dangers and… whether it is actually good for the child or not. So… I feel that the parents who don’t keep up, they have to pull themselves together and start paying attention. (FG2M2)*

In a similar vein, the participants discussed how social media disrupts upbringing. They argued that adolescents lack good role models on social media and that they learn from each other, without supervision or guidance from adults.

*What I fear about social media is that they are lacking role models. Teachers. They learn from each other. And we don’t know what they teach each other. Do you know what I mean? (FG2M1)*

According to the participants, social media has changed how young people communicate, compared to previous generations. Participants felt that adolescents have moved part of their social lives to social media, where they frequently share experiences, thoughts, and feelings with each other. The participants described social interaction on social media as a new language that comes in addition to the oral language, and which they as adults did not understand very well:

*Everything is filtered through [social media], and it comes out in a form that I don’t know very well. It is a completely different language than what we find in the literature, for example. (FG1F1)*

Subtheme 4.3: Wanting to guide adolescents in the use of social media

Several of the participants talked about adolescents as needing guidance regarding social media use, such as raising awareness about body pressure, negative interactions, and other negative influences. Furthermore, it was clear that the participants wanted to understand and help adolescents in their use of social media.

*They need to be a little more critical of what they see and what it does to their own emotions, and become more aware that what they see and the impressions they get can affect them. Either in a positive or in a negative way. We must teach adolescents to become a little more robust, that is, aware of the impressions they get and how it affects the choices they make and what they do with their lives. (FG2F1)*

One participant emphasized that there is no point in asking adolescents not to care about social media or to delete social media platforms, because they have become such an integral part of their lives. Others talked about how teachers and the school must adapt to the new form of interaction that social media constitutes, so that one is better equipped to guide young people in their use.

## 4. Discussion

The aim of this preliminary study was to investigate the perceptions and experiences employees in senior high school have of the relationship between social media and adolescents’ mental health and well-being. The analysis resulted in four themes that provide structure to and framing of what the participants discussed in the focus group interviews. The participants discussed how important social media seemed to be for their pupils (Theme 1), both because social media is a social arena, but also because it is an arena where they get recognition and acceptance from others. They also highlighted community, belonging, openness, and tolerance as positive aspects of social media (Theme 2). Furthermore, the participants emphasized that social media use can have negative consequences for adolescents (Theme 3), both by leading to or exacerbating mental illness, by lowering the quality of communication, and by replacing other important activities or tasks. The fourth and final theme covers a number of opportunities and challenges social media brings into the participants’ roles as school personnel and as parents (Theme 4). The participants expressed an experience of standing on the outside of adolescents’ lives on social media, with limited opportunities to participate in, or to understand, this part of their social world. The participants called for the schools to be better equipped to handle the challenges that arise from social media. This applied both to challenges related to teaching and learning, but also to mental health, where the adolescents, in the eyes of the participants, need guidance about the negative effects of social media and how to cope with the negative aspects of social media.

### 4.1. A Vocal Concern for the Negative Effects of Social Media

Although the participants were asked about both positive and negative aspects of social media use in relation to mental health and well-being among their pupils, negative effects of social media dominated the discussions. Several of the topics discussed by the participants have also been highlighted in the research literature. Under the subtheme “Social media can cause and exacerbate mental health problems” (part of Theme 3), the participants discussed how social media can have a negative effect through social comparison and body pressure. This is in line with an argument put forward in a review by Firth and colleagues [35]: people have an inherent tendency to compare themselves with others, which can become harmful on social media where one is frequently exposed to seemingly “perfect” individuals living successful lives. Quantitative studies have shown that being exposed to ”perfect” body ideals in the mass media can lead to dissatisfaction with one’s own body [36,37,38], which in turn may increase the risk of poor self-esteem and depression [36,39]. Realistic lives, with challenges and hardship are seldom portrayed in social media, and the comparison becomes skewed as adolescents compare both their own negative and positive sides to others “perfect” lives. A qualitative study showed that adolescents view digitally altered images on social media as a cause of negative emotions and low self-esteem [40].

In the review by Firth and colleagues [35], they also pointed out how social media can reinforce negative emotions associated with acceptance and rejection. In the offline world, rejection is often ambiguous and open to interpretation. On social media, on the other hand, social success is quantified through the number of friends, followers, likes, and comments [35]. The participants in this study believed that such feedback has a great impact on how adolescents feel. They also discussed that exclusion is both more visible on social media and seems to be more prevalent due to the absence of adults. Qualitative studies of adolescents have shown that social media can cause them to feel left out, for example by observing that their friends hang out through their posts on social media [24,30]. Although no studies have explicitly investigated whether exclusion/ostracism is more common online than offline, it has been shown that less adult supervision is associated with more bullying [41], and concerns about the lack of online adult supervision has been expressed by several authors (e.g., [42,43]).

The participants in the study considered self-presentation an important motivation for adolescents to use social media, and the participants believed that social media fuels a basic need to get recognition and acceptance from others. The participants argued that self-presentation can be negative in terms of creating a facade and presenting oneself in a way that does not correspond to reality. This is in line with the hypothesis of an “idealized virtual identity”, which claims that people present idealized characteristics on social media that do not necessarily match their real personality [44]. Another hypothesis is the “extended real-life hypothesis”, which states that people use social media to convey their true personality in an extended social context (Back et al., 2010). The way one presents oneself to others varies from person to person [45], where some are more “authentic” than others; they act in line with their natural and unconstructed inclinations, and to a lesser extent in order to make a good impression on others. One can interpret the participants in this study to mean that it is healthier to be authentic than to present oneself in the best possible way on social media. This view is supported by the literature, where being authentic on social media has been linked to higher subjective well-being over time [46]. However, this only applied to those with high subjective well-being in the first place, as current norms of presenting oneself in a positive way prevent those with low subjective well-being from expressing themselves in an authentic way [46]. Another study showed that those who posted status updates with negative content on Facebook were less liked than those who posted positive updates [47], suggesting that authentic self-presentation may be inappropriate on social media if it involves the expression of negativity.

The participants also felt that the introduction of smartphones and social media has led to poorer concentration among pupils. This view is shared by teachers in other countries, where a study from England found that 87 % of teachers agreed with the statement that digital technologies have created an “easily distracted generation with short attention spans” [48]. In support of this view, experimental studies have shown that people learn less if they have access to their phone during a lecture [49,50]. Mendoza and colleagues [50] also demonstrated individual differences when students have access to their phones; those who were easily distracted by text messages received during a lecture remembered less than those who were not distracted by the text messages they received. Additionally, people who scored high on “nomophobia”, defined as a discomfort or fear triggered by not having access to one’s phone [51], remembered less [50]. A related phenomenon, “fear of missing out” (FoMo), may also contribute to excessive phone use and excessive use of social media. FoMo has been defined as “a pervasive apprehension that others might be having rewarding experiences from which one is absent”, and “is characterized by the desire to stay continually connected with what others are doing” ([52], p. 1841). These phenomena may be related to what one of the participants addressed in this study; namely that he feared that social media contributes to creating inequality among pupils, where those who manage to concentrate, despite social media, do better than those who get distracted.

### 4.2. Standing on the Outside

The participants expressed that they find it difficult to understand just how important social media is for the pupils. This can be related directly to a phenomenon discussed in the literature, where young people are “digital natives” (growing up with the internet and social media) and adults are “digital immigrants” (growing up without the internet), which may lead to tension and conflict between them [53,54]. This native versus immigrant divide between adolescents and adults has been criticized for wrongfully assuming that everyone has equal access and opportunity to use digital tools [55]. Still, some form of divide in terms of digital competence and social media use between adults and adolescents is probably evident in countries with high general digital access. In qualitative interviews, adolescents have expressed that adults do not understand their social world and the value of the mobile phone in their lives [22]. The theme “The central role of social media in adolescents’ lives”, nevertheless shows that the participants in the present study had some understanding of the importance of social media in the pupils’ social lives.

The subtheme “Adults experience limited insight into and understanding of adolescents’ social media use” (under Theme 4) also captures that the participants felt that they had little insight into what adolescents do on social media. Several expressed concern that social media is an arena without good mentors and where adults do not have the opportunity to guide and influence their children. Adolescence is characterized by seeking independence and a time where friendships become more important than in preceding developmental stages [56]. Social media can be a reinforcing factor in this process, since adults have limited reach when it comes to moderating and influencing children and adolescents on social media. Social media can contribute to adults losing control over what children and adolescents do and experience in their free time in a different way than when social interaction mainly took place physically and perhaps more openly. One of the participants called for parents to get more involved in what their children are doing on social media. On a slightly different note, the lack of access and insight into what adolescents do on social media, as experienced by the participants in the present study, may contribute to forming a negative view of social media. Adults’ negative reactions to social media use may represent a reaction to a lack of insight into and control over adolescents’ experiences rather than to what actually happens on social media. Studies have indicated, however, that negative experiences online are quite common among children and adolescents. The Norwegian Media Authority [57] recently launched a report showing that 45 % of Norwegian 13–18-year-olds have seen frightening or violent images and hate messages online on one or more occasions. Furthermore, between 30 and 35 % have been exposed to ways to become very thin and ways to injure themselves through social media [57].

### 4.3. Social Media Can Be a Positive Social Arena

As reflected in the subtheme “An important social arena” (under Theme 1), the participants pointed to communication with friends and being part of a community as important motivations among adolescents for using social media. Additionally, social media as a social arena was also what the participants considered a positive aspect of social media. The participants highlighted in particular how young people with low social competence can benefit from social media. Similarly, McKenna, Green, and Gleason [58] showed that those who are anxious in traditional social contexts (face-to-face) can benefit from forming friendships online, where they can express themselves more easily and on their own accord. The authors state that it is easier to open up to others online, and that opening up to others increases perceived intimacy in the relationship. In online social interactions, it is easier to open up because you are more anonymous and the risk of opening up is reduced [58]. In addition, communication via social media often lacks so-called “gating features”—easily observable characteristics such as appearance or poor social skills that may otherwise prevent the establishment of intimacy [58]. The same study also showed that friendships formed through the internet were stable over time, and that they gradually became integrated into the offline social life [58]. In line with this, another study found that students with low self-esteem used social media to expand their network [59]. This indicates that social media can be an important arena for young people to form friendships and to have a fulfilling social life and corresponds to the experiences of the participants in this study.

### 4.4. Implications

The present findings may contribute to a more open discussion about the challenges and opportunities of social media use in schools. The results show that school personnel experience their pupils’ social media use during school hours as challenging. This finding indicates that school personnel have limited resources to deal with the pupils’ social media use, which has implications for schools and the learning environment. On the other hand, our participants recognized positive aspects and opportunities regarding pupils’ social media use that can be utilized for learning purposes. An open dialogue among school personnel and between school personnel and pupils could lead to a more unified approach to phone and social media use during school hours and beyond.

The findings also show that from the perspective of the participants, many adolescents struggle with their social media use and are in need of guidance in order to cope better with the negative sides of social media. One implication of this may be that social media as a phenomenon should be integrated better in health and life skills education, which is part of the core curriculum in Norwegian schools [60]. Social media enables adolescents to stay connected to important others, can provide access to health information, and may enhance learning opportunities [61]; learning to use social media in a sensible and conscious way can be considered a useful life skill.

### 4.5. Strengths and Weaknesses of the Study

The participants were urged to consider their hands-on experiences regarding social media and their pupils’ mental health in the focus group discussions. Nevertheless, it is possible that the participants’ concerns were, at least in part, influenced by the prevailing discourse in society: that social media leads to mental health problems among children and adolescents. Some researchers link the negative focus on social media to a “moral panic” that has repeatedly occurred when new technology becomes widely used in the population [62]. Moral panic refers to “an excessive fear in the population that a given phenomenon will break down society’s morals and norms” ([63], p. 6, translated by the authors). We cannot rule out that the participants were influenced by such moral panic and to a lesser extent by their own experiences or observations. In line with this, the subtheme “Adults experience limited insight into and understanding of adolescents’ use of social media” suggests that the participants’ perspectives were not based on hands-on experiences with, and deep insights into, pupils’ social media use. Furthermore, it is possible that pupils only discuss their social media use with school personnel when they are experiencing problems with their use and to a lesser extent when their experiences are positive or neutral. The participants’ perspectives may still shed light on the role of social media to adolescents’ mental health and well-being.

The present study covers a broad topic and more research is needed to investigate the various subthemes in more detail. Nevertheless, the study provides an overview of the experiences and thoughts that employees in senior high school have regarding the role of social media to the mental health and well-being of their pupils. This perspective is an important piece in understanding how social media can affect mental health and can form the basis for further studies that make the school better equipped to meet the challenges and opportunities that lie in adolescents’ use of social media.

This study is limited in time and space and the results must be interpreted with this in mind. The study included teachers and welfare and pupil support staff from two senior high schools in Bergen municipality in Norway, and the results may not be easily generalized to other parts of the world. Importantly, this preliminary study only included a small number of participants, and larger studies including school personnel from different types of schools, from different counties, and different cultures should be conducted. However, the themes discussed in the present study are reflected in the research literature and it is reasonable to believe that many of the experiences and concerns expressed by the participants also apply elsewhere.

## 5. Conclusions

This study provides an insight into how employees in senior high school assess and experience the relationship between social media and mental health among adolescents. Participants saw social media as an important social arena that can contribute positively in the form of community, belonging, openness, and tolerance and as a tool for communicating with pupils. However, the discussions were also focused on the negative effects of social media, for instance that social media can lead to or exacerbate mental health problems, disturb schoolwork, and impair creativity and social competence. Furthermore, the participants’ discussions reflect the distinction between “digital natives” and “digital immigrants” discussed in the literature. The findings emphasize that social media plays an important and central role in young people’s lives, which to a very high degree takes place in everyday life at school. The participants called for the school to become better equipped to meet the challenges that social media brings, both related to learning and to mental health and life skills.

## Figures and Tables

**Table 1:** Simple overview of the participants. FG = Focus group, F = Female, M = Male.

Participant ID	Focus Group Interview Number	Gender
FG1M1	1	Male
FG1M2	1	Male
FG1M3	1	Male
FG1F1	1	Female
FG1F2	1	Female
FG2M1	2	Male
FG2M2	2	Male
FG2F1	2	Female
FG2F2	2	Female
FG2M3	2	Male
FG2M4	2	Male

## Data Availability

The transcripts of the interviews are not publicly available due to privacy related concerns. Information on transcripts is available from the corresponding author on reasonable request.

## Supplementary Materials

The following are available online at https://www.mdpi.com/article/10.3390/ijerph18179163/s1, Supplementary Material S1: Interview guide. Focus group interview in the project “Health-promoting environment on social media”.
