# Supplementary material for: Pupils’ Use of Social Media and Its Relation to Mental Health from a School Personnel Perspective: A Preliminary Qualitative Study
Source: Int J Environ Res Public Health. 2021 Aug 31;18(17):9163. doi: 10.3390/ijerph18179163 (PMC8431308; doi:10.3390/ijerph18179163)
Supplement: Supplementary file 1 [file ijerph-18-09163-s001.zip › ijerph-1290415-supplementary.pdf]

Interview guide. Focus group interview in the project «Health-promoting environment on social media»

Note: Translated to English by the authors for the purpose of publication. Original version was in Norwegian only.

Introduction (approx. 10 min)

Introduction to the focus group interview:

1. Welcome the participants, and thank them for taking the time to share their thoughts on social media and mental health. Information about the interview, and about us (name, age, role, position).
    a. *Moderator's role:* Present the introduction to the focus group members. Follow the relevant topics that the participants discuss. It will also involve leading the focus group back to relevant topics if the conversation turns to topics that are not central to the issue. (Ensure a safe, welcoming and positive atmosphere). Ensure that the topic of conversation changes when participants begin to run out of things to talk about or begin to repeat themselves (e.g. simply agreeing with each other without adding any new information). Ensure good group dynamics where everyone can have their say.
    b. *The role of the secretary / observer:* Write down and observe what is being discussed. Can act as a co-moderator who can come in and direct the participants back to the topic of the interview guide if necessary.

    The interview is part of Bergen municipality's project "A health-promoting environment on social media". The goal of the project is to develop a health-promoting environment on social media for students in upper secondary school, in collaboration with adolescents. The project is a collaboration between Bergen Municipality, the Norwegian Institute of Public Health and the University College of Western Norway. Much of the media coverage and research on social media addresses the potential negative consequences for mental health. In this interview, we want a more nuanced picture, and want to explore how social media also works as a resource for adolescents. Therefore, we will ask about both negative and positive experiences of social media use among the pupils at your school.

2. Answer any questions about the invitation letter.

3. Practical information: toilets, coffee, water. Break of 10 minutes in the middle, but you have the opportunity to take breaks along the way whenever you want, without explanations or asking for permission.

4. Inform about focus group interviews as a method of collecting data. A focus group interview is a structured group interview where the main purpose is the exchange of experiences, opinions and views between the participants and in this way develop knowledge.

5. Review guidelines for the focus group interview:

    a. There are no right and wrong answers, just different opinions or views.
    b. One person speaks at a time
    c. You do not have to agree with others, but you must listen with respect to what others say.
    d. Our role is to guide you through the discussion.
    e. Please talk to each other and not to us.
    f. Please put your phones in flight mode

6. Information about our duty of confidentiality as healthcare professionals. We cannot impose a duty of confidentiality on participants in the same way, but we strongly encourage respect and the "duty of confidentiality" towards participants and the information that emerges during the interview. In order to have a good discussion, it is important that everyone feels safe to share, and that what is said here stays between us here in this room.

7. We appreciate and are interested in each of your individual views, experiences and opinions. Regardless of how the questions are formulated and what we ask, it is up to you to consider what and how much you want to share in the group.

8. Ask them to read through and sign the consent form if they agree to participate. Answer any questions.

9. Ask explicitly about permission to record the interview. Then start audio recording.

10. Presentation round of the participants, where they present first name and which subjects they teach or if they work as pupil support staff

11. Repeat the purpose of the study: what do you think about social media and how the use of social media is related to adolescents’ lives and mental health.

12. Finally, there will be a brief description of terms used. In this focus group interview;
    a. we have chosen to use ***social media*** as a collective term for all two-way communication that takes place over the internet, websites and apps (e.g., blog, chats, multiplayer online gaming which include communication, Twitter, Instagram, Snapchat, Facebook, Messenger , YouTube, etc.)
    b. ***Mental health*** can be both good and bad. Poor mental health can involve mental disorders such as anxiety and depression, but also other symptoms/conditions such as stress, difficulty sleeping and concentration

problems. Good mental health can be experiencing happiness, feeling that your life is meaningful, having good friends, energy, self-esteem or feeling happy with life.

Opening question (approx. 10 min)

1. Can you briefly tell us what you think or know that adolescents use social media for?

The main part (approx. 30/40 min)

2. How do you experience that social media can be a positive factor in adolescents' lives?

Instructions for the moderator: We are looking for their own experiences with this, rather than how they think or have heard it can be.

If necessary, repeat what we mean by mental health. Both good and bad mental health.

Follow-up questions:
What do you think that social media means to adolescents? Can you say something about what you think makes social media important adolescents, if it is?

Social media allow for a range of activities, such as sharing content, commenting / discussing or just looking at things. Which activities do you think foster good mental health?

Do you think that how adolescents feel affects their use of social media?

What other things are adolescents doing on social media that you think are positive for them, beyond the purely practical?

2b. What do you think that adolescents would have missed during a day without access to social media?

3. How do you experience that social media can be a negative factor in adolescents'

lives?

Instructions for the moderator: We are looking for their own experiences with this, not how they think or have heard it can be.

If necessary, repeat what we mean by mental health. Both good and bad mental health.

Follow-up questions:
How can social media lead to poor mental health?

What features on social media can make adolescents feel bad?

Which social media features do you like the least?

Do you think that how adolescents feel affects their use social media?

Final questions (approx. 10 min)

4. Which of the topics that we have talked about today do you think are particularly important and central?

5. Is there anything we have not talked about in this interview that you think is important to include about social media and mental health?

Conclusion

1. Thank you for participating and for your contribution to knowledge development.
2. Remind of the duty of confidentiality and mutual respect for each other.
